# 2,3-(3,6,9-Trioxaundecane-1,11-diyl­disulfan­yl)-1,4,5,8-tetra­thia­fulvalene-6,7-dicarbonitrile

**DOI:** 10.1107/S1600536810017587

**Published:** 2010-05-19

**Authors:** Rui-Bin Hou, Bao Li, Tie Chen, Bing-Zhu Yin, Li-Xin Wu

**Affiliations:** a Key Laboratory of Organism Functional Factors of the Changbai Mountain, Yanbian University, Ministry of Education, Yanji 133002, People’s Republic of China; b State Key Laboratory of Supramolecular Structure and Materials, College of Chemistry, Jilin University, Changchun 130012, People’s Republic of China

## Abstract

In the title compound, C_16_H_16_N_2_O_3_S_6_, the two five-membered rings form a dihedral angle of 7.86 (9)°. Weak C—H⋯N hydrogen bonds link the mol­ecules to form a chain along *c*; the chains are further connected by weak C—H⋯O hydrogen bonds to form a three-dimensional supra­molecular network.

## Related literature

For background to the use of dithiacrown ether annulated tetrathiafulvalenes as sensor molecules for various metal cations, see Moore *et al.* (2000 ▶); Otsubo & Ogura (1985 ▶). For the synthesis, see Yin *et al.* (2006 ▶). For a related structure, see Hou *et al.* (2009 ▶).
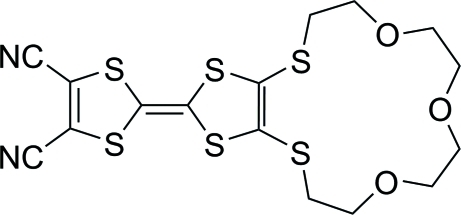


## Experimental

### 

#### Crystal data

C_16_H_16_N_2_O_3_S_6_
*M* *_r_* = 476.67
Triclinic, 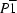

*a* = 8.300 (5) Å
*b* = 9.186 (5) Å
*c* = 13.892 (10) Å
α = 100.42 (3)°
β = 92.31 (3)°
γ = 95.60 (2)°
*V* = 1035.0 (11) Å^3^
*Z* = 2
Mo *K*α radiation
μ = 0.68 mm^−1^
*T* = 290 K
0.13 × 0.12 × 0.10 mm

#### Data collection

Rigaku R-AXIS RAPID diffractometer
Absorption correction: multi-scan (*ABSCOR*; Higashi, 1995 ▶) *T*_min_ = 0.917, *T*_max_ = 0.935
10214 measured reflections
4702 independent reflections
3936 reflections with *I* > 2σ(*I*)
*R*_int_ = 0.026

#### Refinement

*R*[*F* ^2^ > 2σ(*F* ^2^)] = 0.032
*wR*(*F* ^2^) = 0.088
*S* = 1.07
4702 reflections
245 parameters
H-atom parameters constrained
Δρ_max_ = 0.52 e Å^−3^
Δρ_min_ = −0.31 e Å^−3^

### 

Data collection: *RAPID-AUTO* (Rigaku, 1998 ▶); cell refinement: *RAPID-AUTO*; data reduction: *CrystalStructure* (Rigaku/MSC, 2002 ▶); program(s) used to solve structure: *SHELXS97* (Sheldrick, 2008 ▶); program(s) used to refine structure: *SHELXL97* (Sheldrick, 2008 ▶); molecular graphics: *PLATON* (Spek, 2009 ▶); software used to prepare material for publication: *SHELXL97*.

## Supplementary Material

Crystal structure: contains datablocks global, I. DOI: 10.1107/S1600536810017587/ng2773sup1.cif

Structure factors: contains datablocks I. DOI: 10.1107/S1600536810017587/ng2773Isup2.hkl

Additional supplementary materials:  crystallographic information; 3D view; checkCIF report

## Figures and Tables

**Table 1:** Hydrogen-bond geometry (Å, °)

*D*—H⋯*A*	*D*—H	H⋯*A*	*D*⋯*A*	*D*—H⋯*A*
C13—H13*A*⋯N1^i^	0.97	2.65	3.534 (3)	152
C14—H14*A*⋯N1^ii^	0.97	2.74	3.691 (3)	168
C14—H14*B*⋯O1^iii^	0.97	2.64	3.401 (3)	136
C9—H9*A*⋯O2^iv^	0.97	2.57	3.406 (3)	144
C15—H15*B*⋯O3^v^	0.97	2.47	3.317 (2)	146

Symmetry codes: (i) 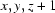; (ii) 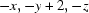; (iii) 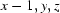; (iv) 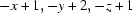; (v) 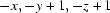.

**Fig. 1.**
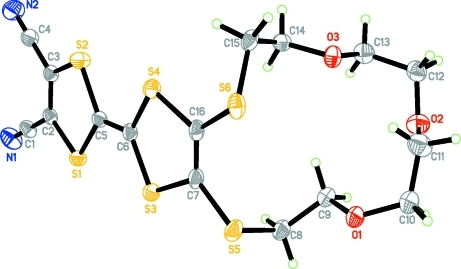
The asymmetric unit of the title compound, with the atom numbering. Displacement ellipsoids of non-H atoms are drawn at the 30% probalility level.

## supplementary crystallographic information

### Comment

Dithiacrown ether annulated tetrathiafulvalenes have received great attentions as sensors molecule for various metal cations (Otsubo *et al.*, 1985; Moore *et al.*, 2000). These sensors can recognize selectively the virous metal cations to singel electrochemical information. We incorpolated TTF with a 15-membered O, S hybrid crown ether to synthesize the title compound because it should be able to bind sodium ion (Yin *et al.*, 2006). We report herein the synthesis and structure of the title compound.

The title compound, (I), as shown in Fig. 1, all bond lengths and angles are normal and comparable with those reported for the related structure (Hou *et al.*, 2009). In the crystal, weak C—H···O hydrogen bonds (table 1) link the molecules into two-dimensional network in ac plane. The crystal structure is further stablized by weak C—H···N hydrogen bonds along c dirction.

### Experimental

The title compound, (I), was prepared according to literature (Yin *et al.*, 2006) and single crystals suitable for X-ray diffraction were prepared by slow evaporation a mixture of dichloromethane and petroleum (60-90 °C) at room temperature.

### Refinement

Carbon-bound H-atoms were placed in calculated positions with C—H = 0.97 A and were included in the refinement in the riding model with U_iso_(H) = 1.2 U_eq_(C).

### Crystal data

**Table:**

C_16_H_16_N_2_O_3_S_6_	*Z* = 2
*M**_r_* = 476.67	*F*(000) = 492
Triclinic, *P*1	*D*_x_ = 1.530 Mg m^−^^3^
Hall symbol: -P 1	Mo *K*α radiation, λ = 0.71073 Å
*a* = 8.300 (5) Å	Cell parameters from 8487 reflections
*b* = 9.186 (5) Å	θ = 3.2–27.5°
*c* = 13.892 (10) Å	µ = 0.68 mm^−^^1^
α = 100.42 (3)°	*T* = 290 K
β = 92.31 (3)°	Block, black
γ = 95.60 (2)°	0.13 × 0.12 × 0.10 mm
*V* = 1035.0 (11) Å^3^	

### Data collection

**Table:**

Rigaku R-AXIS RAPID diffractometer	4702 independent reflections
Radiation source: fine-focus sealed tube	3936 reflections with *I* > 2σ(*I*)
graphite	*R*_int_ = 0.026
ω scans	θ_max_ = 27.5°, θ_min_ = 3.2°
Absorption correction: multi-scan (*ABSCOR*; Higashi, 1995)	*h* = −10→10
*T*_min_ = 0.917, *T*_max_ = 0.935	*k* = −10→11
10214 measured reflections	*l* = −18→18

### Refinement

**Table:**

Refinement on *F*^2^	Secondary atom site location: difference Fourier map
Least-squares matrix: full	Hydrogen site location: inferred from neighbouring sites
*R*[*F*^2^ > 2σ(*F*^2^)] = 0.032	H-atom parameters constrained
*wR*(*F*^2^) = 0.088	*w* = 1/[σ^2^(*F*_o_^2^) + (0.0449*P*)^2^ + 0.1948*P*] where *P* = (*F*_o_^2^ + 2*F*_c_^2^)/3
*S* = 1.07	(Δ/σ)_max_ = 0.001
4702 reflections	Δρ_max_ = 0.52 e Å^−^^3^
245 parameters	Δρ_min_ = −0.30 e Å^−^^3^
0 restraints	Extinction correction: *SHELXL97* (Sheldrick, 2008), Fc^*^=kFc[1+0.001xFc^2^λ^3^/sin(2θ)]^-1/4^
Primary atom site location: structure-invariant direct methods	Extinction coefficient: 0.027 (2)

### Special details

**Table:**

Experimental. (See detailed section in the paper)
Geometry. All esds (except the esd in the dihedral angle between two l.s. planes) are estimated using the full covariance matrix. The cell esds are taken into account individually in the estimation of esds in distances, angles and torsion angles; correlations between esds in cell parameters are only used when they are defined by crystal symmetry. An approximate (isotropic) treatment of cell esds is used for estimating esds involving l.s. planes.
Refinement. Refinement of *F*^2^ against ALL reflections. The weighted *R*-factor wR and goodness of fit *S* are based on *F*^2^, conventional *R*-factors *R* are based on *F*, with *F* set to zero for negative *F*^2^. The threshold expression of *F*^2^ > σ(*F*^2^) is used only for calculating *R*-factors(gt) etc. and is not relevant to the choice of reflections for refinement. *R*-factors based on *F*^2^ are statistically about twice as large as those based on *F*, and *R*- factors based on ALL data will be even larger.

### Fractional atomic coordinates and isotropic or equivalent isotropic displacement parameters (Å^2^)

**Table:**

	*x*	*y*	*z*	*U*_iso_*/*U*_eq_	
C1	−0.1516 (2)	0.9624 (2)	−0.20634 (12)	0.0455 (4)	
C2	−0.1520 (2)	0.88171 (17)	−0.12738 (11)	0.0364 (3)	
C3	−0.2869 (2)	0.84467 (18)	−0.08319 (11)	0.0377 (3)	
C4	−0.4436 (2)	0.8829 (2)	−0.10925 (13)	0.0493 (4)	
C5	−0.05873 (19)	0.74212 (17)	0.00468 (11)	0.0353 (3)	
C6	0.02801 (19)	0.68050 (18)	0.06787 (11)	0.0374 (3)	
C7	0.2533 (2)	0.60022 (18)	0.17572 (11)	0.0406 (4)	
C8	0.5249 (2)	0.7500 (2)	0.28490 (13)	0.0470 (4)	
H8A	0.6417	0.7629	0.2811	0.056*	
H8B	0.4803	0.8258	0.2548	0.056*	
C9	0.4861 (2)	0.77382 (19)	0.39115 (12)	0.0428 (4)	
H9A	0.5244	0.8748	0.4233	0.051*	
H9B	0.3699	0.7581	0.3967	0.051*	
C10	0.5910 (3)	0.7045 (3)	0.53927 (15)	0.0621 (5)	
H10A	0.6581	0.7990	0.5566	0.075*	
H10B	0.6520	0.6293	0.5596	0.075*	
C11	0.4441 (3)	0.7141 (2)	0.59613 (16)	0.0625 (5)	
H11A	0.3571	0.6423	0.5626	0.075*	
H11B	0.4667	0.6906	0.6603	0.075*	
C12	0.2389 (2)	0.8716 (2)	0.64099 (13)	0.0506 (4)	
H12A	0.2396	0.9632	0.6884	0.061*	
H12B	0.2088	0.7892	0.6738	0.061*	
C13	0.1159 (2)	0.87039 (19)	0.55895 (14)	0.0499 (4)	
H13A	0.0166	0.9038	0.5853	0.060*	
H13B	0.1566	0.9378	0.5171	0.060*	
C14	−0.0256 (2)	0.71693 (19)	0.42162 (12)	0.0401 (4)	
H14A	0.0240	0.7710	0.3747	0.048*	
H14B	−0.1227	0.7616	0.4424	0.048*	
C15	−0.0681 (2)	0.55669 (19)	0.37564 (12)	0.0434 (4)	
H15A	−0.1587	0.5490	0.3280	0.052*	
H15B	−0.1029	0.5019	0.4260	0.052*	
C16	0.1146 (2)	0.56287 (17)	0.21572 (11)	0.0385 (4)	
N1	−0.1521 (3)	1.0257 (2)	−0.26936 (13)	0.0688 (5)	
N2	−0.5676 (2)	0.9136 (3)	−0.12990 (15)	0.0767 (6)	
O1	0.56498 (15)	0.67023 (14)	0.43571 (9)	0.0494 (3)	
O2	0.39558 (16)	0.85988 (14)	0.60659 (10)	0.0540 (3)	
O3	0.08385 (15)	0.72386 (12)	0.50341 (8)	0.0419 (3)	
S1	0.02900 (5)	0.82515 (5)	−0.08826 (3)	0.04072 (12)	
S2	−0.27073 (5)	0.74152 (6)	0.00882 (3)	0.04764 (13)	
S3	0.23772 (5)	0.67410 (5)	0.06743 (3)	0.04530 (12)	
S4	−0.06611 (5)	0.59369 (5)	0.15614 (3)	0.04415 (12)	
S5	0.44704 (6)	0.56746 (5)	0.21477 (4)	0.05140 (14)	
S6	0.09787 (6)	0.47074 (5)	0.31522 (3)	0.04736 (13)	

### Atomic displacement parameters (Å^2^)

**Table:**

	*U*^11^	*U*^22^	*U*^33^	*U*^12^	*U*^13^	*U*^23^
C1	0.0563 (10)	0.0477 (9)	0.0365 (8)	0.0147 (8)	0.0069 (7)	0.0124 (7)
C2	0.0451 (8)	0.0380 (8)	0.0278 (7)	0.0106 (7)	0.0008 (6)	0.0076 (6)
C3	0.0410 (8)	0.0444 (8)	0.0301 (7)	0.0123 (7)	−0.0020 (6)	0.0100 (6)
C4	0.0463 (10)	0.0674 (11)	0.0386 (9)	0.0175 (9)	0.0003 (7)	0.0160 (8)
C5	0.0369 (8)	0.0431 (8)	0.0279 (7)	0.0110 (7)	0.0006 (6)	0.0083 (6)
C6	0.0407 (8)	0.0448 (8)	0.0274 (7)	0.0113 (7)	−0.0024 (6)	0.0064 (6)
C7	0.0447 (9)	0.0432 (8)	0.0333 (8)	0.0142 (7)	−0.0099 (7)	0.0031 (6)
C8	0.0429 (9)	0.0542 (10)	0.0452 (9)	0.0016 (8)	−0.0058 (7)	0.0167 (8)
C9	0.0398 (8)	0.0426 (9)	0.0456 (9)	0.0028 (7)	−0.0038 (7)	0.0097 (7)
C10	0.0551 (11)	0.0863 (15)	0.0479 (11)	0.0191 (11)	−0.0054 (9)	0.0160 (10)
C11	0.0723 (14)	0.0645 (12)	0.0598 (12)	0.0214 (11)	0.0145 (10)	0.0259 (10)
C12	0.0559 (11)	0.0452 (9)	0.0458 (10)	0.0001 (8)	0.0052 (8)	−0.0024 (8)
C13	0.0561 (11)	0.0388 (9)	0.0544 (10)	0.0115 (8)	0.0050 (8)	0.0037 (8)
C14	0.0381 (8)	0.0480 (9)	0.0399 (8)	0.0100 (7)	0.0039 (6)	0.0202 (7)
C15	0.0413 (8)	0.0516 (9)	0.0391 (8)	−0.0083 (7)	−0.0085 (7)	0.0230 (7)
C16	0.0477 (9)	0.0382 (8)	0.0299 (7)	0.0131 (7)	−0.0096 (7)	0.0048 (6)
N1	0.0948 (14)	0.0705 (11)	0.0523 (10)	0.0233 (10)	0.0155 (9)	0.0307 (9)
N2	0.0525 (10)	0.1179 (17)	0.0673 (12)	0.0318 (11)	−0.0040 (9)	0.0268 (11)
O1	0.0477 (7)	0.0621 (8)	0.0408 (6)	0.0152 (6)	−0.0050 (5)	0.0127 (6)
O2	0.0509 (7)	0.0463 (7)	0.0644 (8)	0.0010 (6)	0.0077 (6)	0.0103 (6)
O3	0.0508 (7)	0.0355 (6)	0.0399 (6)	0.0046 (5)	−0.0055 (5)	0.0104 (5)
S1	0.0380 (2)	0.0521 (2)	0.0354 (2)	0.01113 (18)	0.00434 (16)	0.01327 (17)
S2	0.0403 (2)	0.0657 (3)	0.0470 (2)	0.0160 (2)	0.00757 (18)	0.0305 (2)
S3	0.0407 (2)	0.0629 (3)	0.0344 (2)	0.0145 (2)	−0.00208 (16)	0.01077 (19)
S4	0.0418 (2)	0.0618 (3)	0.0331 (2)	0.01220 (19)	−0.00430 (16)	0.01804 (18)
S5	0.0472 (3)	0.0551 (3)	0.0507 (3)	0.0212 (2)	−0.0144 (2)	0.0019 (2)
S6	0.0675 (3)	0.0392 (2)	0.0371 (2)	0.0133 (2)	−0.0134 (2)	0.01131 (17)

### Geometric parameters (Å, °)

**Table:**

C1—N1	1.135 (2)	C10—C11	1.480 (3)
C1—C2	1.430 (2)	C10—H10A	0.9700
C2—C3	1.343 (2)	C10—H10B	0.9700
C2—S1	1.7352 (18)	C11—O2	1.420 (3)
C3—C4	1.430 (2)	C11—H11A	0.9700
C3—S2	1.7318 (18)	C11—H11B	0.9700
C4—N2	1.133 (3)	C12—O2	1.411 (2)
C5—C6	1.348 (2)	C12—C13	1.497 (3)
C5—S1	1.7615 (18)	C12—H12A	0.9700
C5—S2	1.7625 (19)	C12—H12B	0.9700
C6—S3	1.7476 (19)	C13—O3	1.420 (2)
C6—S4	1.7490 (19)	C13—H13A	0.9700
C7—C16	1.340 (3)	C13—H13B	0.9700
C7—S5	1.7479 (19)	C14—O3	1.414 (2)
C7—S3	1.764 (2)	C14—C15	1.496 (2)
C8—C9	1.505 (3)	C14—H14A	0.9700
C8—S5	1.823 (2)	C14—H14B	0.9700
C8—H8A	0.9700	C15—S6	1.814 (2)
C8—H8B	0.9700	C15—H15A	0.9700
C9—O1	1.421 (2)	C15—H15B	0.9700
C9—H9A	0.9700	C16—S6	1.7499 (19)
C9—H9B	0.9700	C16—S4	1.7555 (18)
C10—O1	1.419 (2)		
			
N1—C1—C2	179.5 (2)	C10—C11—H11B	109.7
C3—C2—C1	123.03 (15)	H11A—C11—H11B	108.2
C3—C2—S1	118.17 (13)	O2—C12—C13	111.50 (16)
C1—C2—S1	118.78 (13)	O2—C12—H12A	109.3
C2—C3—C4	123.67 (16)	C13—C12—H12A	109.3
C2—C3—S2	118.12 (12)	O2—C12—H12B	109.3
C4—C3—S2	118.19 (14)	C13—C12—H12B	109.3
N2—C4—C3	179.9 (3)	H12A—C12—H12B	108.0
C6—C5—S1	123.07 (13)	O3—C13—C12	109.47 (15)
C6—C5—S2	121.53 (13)	O3—C13—H13A	109.8
S1—C5—S2	115.39 (9)	C12—C13—H13A	109.8
C5—C6—S3	124.32 (14)	O3—C13—H13B	109.8
C5—C6—S4	121.23 (14)	C12—C13—H13B	109.8
S3—C6—S4	114.42 (9)	H13A—C13—H13B	108.2
C16—C7—S5	125.91 (13)	O3—C14—C15	108.09 (13)
C16—C7—S3	117.11 (12)	O3—C14—H14A	110.1
S5—C7—S3	116.72 (11)	C15—C14—H14A	110.1
C9—C8—S5	114.18 (13)	O3—C14—H14B	110.1
C9—C8—H8A	108.7	C15—C14—H14B	110.1
S5—C8—H8A	108.7	H14A—C14—H14B	108.4
C9—C8—H8B	108.7	C14—C15—S6	113.74 (12)
S5—C8—H8B	108.7	C14—C15—H15A	108.8
H8A—C8—H8B	107.6	S6—C15—H15A	108.8
O1—C9—C8	107.67 (14)	C14—C15—H15B	108.8
O1—C9—H9A	110.2	S6—C15—H15B	108.8
C8—C9—H9A	110.2	H15A—C15—H15B	107.7
O1—C9—H9B	110.2	C7—C16—S6	125.35 (13)
C8—C9—H9B	110.2	C7—C16—S4	116.95 (13)
H9A—C9—H9B	108.5	S6—C16—S4	117.42 (11)
O1—C10—C11	116.36 (18)	C10—O1—C9	116.55 (15)
O1—C10—H10A	108.2	C12—O2—C11	113.79 (15)
C11—C10—H10A	108.2	C14—O3—C13	112.15 (13)
O1—C10—H10B	108.2	C2—S1—C5	94.00 (8)
C11—C10—H10B	108.2	C3—S2—C5	94.10 (8)
H10A—C10—H10B	107.4	C6—S3—C7	95.23 (8)
O2—C11—C10	109.80 (18)	C6—S4—C16	95.58 (9)
O2—C11—H11A	109.7	C7—S5—C8	101.42 (9)
C10—C11—H11A	109.7	C16—S6—C15	100.76 (8)
O2—C11—H11B	109.7		

### Hydrogen-bond geometry (Å, °)

**Table:**

*D*—H···*A*	*D*—H	H···*A*	*D*···*A*	*D*—H···*A*
C13—H13A···N1^i^	0.97	2.65	3.534 (3)	152
C14—H14A···N1^ii^	0.97	2.74	3.691 (3)	168
C14—H14B···O1^iii^	0.97	2.64	3.401 (3)	136
C15—H15A···O1^iii^	0.97	2.99	3.402 (3)	107
C9—H9A···O2^iv^	0.97	2.57	3.406 (3)	144
C15—H15B···O3^v^	0.97	2.47	3.317 (2)	146

Symmetry codes: (i) *x*, *y*, *z*+1; (ii) −*x*, −*y*+2, −*z*; (iii) *x*−1, *y*, *z*; (iv) −*x*+1, −*y*+2, −*z*+1; (v) −*x*, −*y*+1, −*z*+1.
